# Symptomatology of the Pupil*Read before the Buffalo Medical Club April 12, 1882.

**Published:** 1882-05

**Authors:** Lucien Howe


### BUFFALO

### Medical and Surgical Journal.

Vol. XXI. MAY, 1882. No. 10.

### Original Communications.

### Symptomatology of the Pupil.*

By Lucien Howe, M. D.

PART. I.—PHYSIOLOGICAL CHANGES.

Unless a writer has a special claim to originality in the subject which he proposes to present, it is becoming that he should give some reason for adding to an already abundant and almost overburdened mass of medical literature. But as long as our knowledge of any topic has not yet crystallized into definite form—while the facts known concerning it are still somewhat disconnected—and especially while new observations are being continually made; during this time, any brief and systematic arrangement of these facts is usually acceptable to a large class of readers.

I shall endeavor, therefore, to present here a brief résumé of our present knowledge of the subject under consideration, together with some observations of my own, which seemed to be of value, with the hope that this will be acceptable to those practitioners who seek principally for truths which can be applied clinically.

* Read before the Buffalo Medical Club April 12, 1882.

Concerning the movements of the iris, we have, unfortunately, very, very much yet to learn, and it is not a little remarkable that the causes of phenomena, which are so readily observed, which have been studied so long and constantly, and withal are so full of importance—that these should still be involved in so much obscurity.

As we proceed to our study, we naturally begin by examining the structure of the iris. We know, of course, that it is a diaphragm placed in the anterior part of the eye. It might be interesting to consider the layers of epithelium which cover its two surfaces, its thick deposit of pigment posteriorly, or the numerous vessels which ramify throughout the entire structure. But the portion of special interest to us, at present, is its muscular tissue and the nerves which supply it. The muscular fibers are arranged in two sets. One, a comparatively large circular band which surrounds the edge of the pupil, is called the *sphincter pupillæ*. The other—more indistinct—is composed of radiating fibers, which pass from the sphincter outwards toward the peripheral edge [44] *, and these together, are known as the *dilator pupillæ*. The first may be compared to the hub of a wheel, and the second to its spokes. The opposing action of these two separate sets of fibers naturally suggests itself, for contraction of either one, is necessarily accompanied by relaxation of the other.

It is worthy of note that the existence of distinct radial fibers was not so early recognized as were the circular ones, and several eminent authorities in microscopical anatomy, or in physiology, have joined in the controversy concerning them. On the one side we find that Brucke [38] affirmed their existence, while Henle [52] and other equally acute observers, either denied their presence in the human subject or considered it uncertain. This difference of opinion, formerly held, showed at least how minute and radial fibers are, and how intimately they are interwoven with the blood vessels, although at present we are in a position to speak of them without hesitation.

* The numbers refer to a list of references which will accompany Part II in the next issue.

Having thus glanced at the two muscles which contract and dilate the pupil, let us next trace the course of the nerves which supply them. These come principally [75]; first, from the motor oculi, second from the sympathetic, and third from the ophthalmic branch of the trifacial. The general positions of these are too well known to require extended description, but their relations in many pathological conditions are so important, that I shall not hesitate to rehearse familiar detail, rather than pass lightly over so important a part of the subject. Moreover, I would venture to suggest to any one whose recollection of any of the particulars concerning the cranial nerves, has begun to fade into a myth of the dissecting room, that he take down his copy of Gray or Wilson and review the portions under consideration.

Turning our attention first to the motor oculi, it will be remembered that the fibers of this nerve arise near the motor tract in the pons varolii, and emerge from the brain just in front of the pons and to the inner side of the crus cerebri. Thence it passes forward through a sheath in the dura mater toward the orbit. Indeed, it enters that sheath, so soon after leaving the brain, that it is followed with some little difficulty when we remove that organ from the skull, and it should not be forgotten that the protection thus afforded, renders it less liable to lesions in the cranial part of its course than when nearer its origin, or when in the orbit. While in this sheath, it passes very near to the sixth nerve which is on its way to the external rectus muscle, and also almost touches the sinus cavernosus. Any simultaneous disturbance of function, therefore, of the external, and of the other recti muscles, or of these, with any evidences of trouble in the sinus mentioned, would of course indicate the locality of the lesion. After entering the orbit, the motor oculi gives off branches to all the ocular muscles—with the exception of the superior oblique and external rectus—and also a small twig, but a very important one in this connection, which goes to the ophthalmic ganglion.

We shall presently find that the nerve just described supplies the sphincter muscle of the iris, and as its function is opposed to one which supplies the radial fibers, it is but natural that we should regard next the branches of the sympathetic. It is well known that the intricate interlacing [101] of these fibers almost defies description, and indeed they are liable to so many variations at least in the region under consideration, that no detailed account of their course is necessary. For our present purpose, it is sufficient to recall the general positions and relations of the three cervical ganglia [52] with their communications, and especially that group of fibers which, lying just by the side of the sella turcica is called the cavernous plexus. It communicates with the third, fourth and sixth nerves and gives off one branch of importance which goes to the ophthalmic ganglion. This ganglion receives a twig from a third source, namely, from a branch of the trigeminus, but that being a nerve of sensation is not of so great importance in a study of the motions of the iris.

The ophthalmic ganglion is seldom seen in the dissecting room, but if the outer and upper wall of the orbit have been removed, and the external rectus laid bare and then divided, a little further patience will enable one to find the three branches which go to form that small reddish-brown enlargement just at the outer side of the optic nerve. Its half dozen or more branches of distribution can also be seen, as they pass forward to pierce the posterior part of the sclerotic, whence they find their way to the iris. The filaments connected with the motor-oculi are distributed ultimately to the sphincter muscle, those from the sympathetic go to its radial fibers, and those from the trigeminus to its entire structure [6] [67].

Our knowledge of the action of these three nerves upon the iris, is derived principally from experiment, but also from clinical observations and post-mortem appearances. As for the facts learned by experiment, we must exercise a certain amount of caution in making deductions from the phenomena observed. Thus, the experiment itself, may introduce complicating factors, and besides the conclusion which could properly be drawn as regards an animal, might not hold good when applied to the human species. These experiments are of various kinds, and much has undoubtedly been learned from the use of mydriatics and myotics, but it is principally from the effects following the division of the sympathetic or motor oculi, or their irritation by electricity, that we must rely for our knowledge. Making allowance, however, for possibilities of error—which, of course, are always present in problems of this sort—irrespective of the source from which the information is gained, we are safe in asserting that the filaments of the trigeminus are principally for sensation;

That contraction of the pupil or myosis is caused

1st. By irritation of the motor oculi, or

2d. By paralysis of the sympathetic.

And dilatation of the pupil or mydriasis is caused

1st. By irritation of the sympathetic, or

2d. By paralysis of the motor oculi.

A moment's reflection will show the reason of this important relation. For, as the sphincter fibers at the pupillary edge of the iris, and its radiating fibers, are direct opponents of each other, it is evident that the same effect can be produced by contracting one set or relaxing the other. A small pupil may result *either* from a forcible contraction of the sphincter, *or*, from unusual relaxation of its opponent—the radial fibers. Conversely, a large pupil may result *either* from a forcible contraction of the radial fibers, *or*, from unusual relaxation of their opponent—the sphincter. The fact of this relation is sufficiently important to be worthy of reiteration.

The usual size of the pupil may be varied within physiological limits by three causes. These are: 1st, The age of the individual; 2d, Contraction in the effort of accommodation; 3d, Contraction due to increased intensity of light. As middle life is passed and old age advances, the pupil becomes noticably smaller than in youth. One of the most important factors in the production of this contraction is probably an increased effort in the act of accommodation, necessitated by the gradual hardening of the crystalline lens.

In considering the character of this muscular action, we are naturally led to the second physiological variation, namely, that which occurs in the act of accommodation. It is now generally conceded, that when the eye regards a distant object, it is in a state of rest, but the nearer the object approaches, the greater is the effort made to see it distinctly, or, in other words, to keep the image on the retina. In this act of accommodation the pupil becomes perceptibly smaller [15].

This can be noticed in the eye of a person who looks alternately at an object distant twenty feet or more, and at a point only a few inches in front of the face. It can be seen, however, with even greater ease, if the one whose eye is observed, happens to possess the power of voluntarily adjusting the focus and relaxing the effort. While experimenting thus upon the print of a book before him the varying size of his pupils is readily observed. Not only do they contract in the act of accommodation, but it has been noticed that persons, like watchmakers, jewelers and others, who are accustomed to use their eyes for close work during a long time, are apt to have that aperture unusually small, and the contraction is occasionally accompanied by such symptoms of irritation, as to simulate a real inflammation.

Let us next turn our attention to the changes which the pupil undergoes from the influence of light. This contraction may be the result of the rays entering one eye directly, or may come about indirectly through the impression produced on the other. It is not known exactly what the connection is between the two eyes, upon which this relation in every way depends, [1], but certain it is, that the retina, optic nerve and optic tract of the one into which the light falls, must be in a healthy condition in order to have the reaction take place. In proportion as the perceptive and conductive portions of the nerves are imperfect, in that proportion does the pupil generally [34] remain sluggish. Moreover, the older the person is, the less apparent is this effect of the impact of light, and the entire cessation of the reaction after death, is of importance, when a question arises in regard to recognizing that condition [64].

The reaction is easily shown, as we know by closing one eye entirely, and then shading its fellow for a moment or two with the palm of the hand. On removing the hand suddenly, the pupil of a healthy eye at once contracts. When making this observation clinically, it is always well to repeat it with each eye separately, in order to be assured that the conditions are the same on both sides. It should finally be noted that the reaction of the pupil is due to the impact of light on the retina, and not on the iris. If the rays from a flame be converged by means of a small magnifying glass upon the iris, little or no effect is produced; but if the light be then allowed to enter the pupil, contraction at once follows.

The degree of contraction varies in proportion to the intensity of light, and I may remark in passing, that as the electric light is one of the strongest known, with the exception of sunlight, that the maximum amount of contraction is produced by it. But this of itself, is of no special detriment, and as it is the only questionable effect of that source of illumination, we must regard the popular objections to the electric light as entirely unfounded.

The importance of recognizing clinically these physiological variations, is too evident to require more than simple mention. As the physician approaches the bedside of a patient who is perhaps speechless or unconscious, and then observes the size and movements of the pupil, he should remember what condition to expect from the age of the patient, he should consider the distance at which objects are observed, and the relation between the age and degree of reaction from light.

